# ATM kinase inhibitor AZD0156 in combination with irinotecan and 5-fluorouracil in preclinical models of colorectal cancer

**DOI:** 10.1186/s12885-022-10084-7

**Published:** 2022-10-29

**Authors:** S. Lindsey Davis, Sarah J. Hartman, Stacey M. Bagby, Marina Schlaepfer, Betelehem W. Yacob, Tonia Tse, Dennis M. Simmons, Jennifer R. Diamond, Christopher H. Lieu, Alexis D. Leal, Elaine B. Cadogan, Gareth D. Hughes, Stephen T. Durant, Wells A. Messersmith, Todd M. Pitts

**Affiliations:** 1 University of Colorado Cancer Center, Aurora, CO USA; 2 AstraZeneca, Cambridge, UK

**Keywords:** ATM, Colorectal cancer, DNA damage repair, AZD0156, Irinotecan

## Abstract

**Background:**

AZD0156 is an oral inhibitor of ATM, a serine threonine kinase that plays a key role in DNA damage response (DDR) associated with double-strand breaks. Topoisomerase-I inhibitor irinotecan is used clinically to treat colorectal cancer (CRC), often in combination with 5-fluorouracil (5FU). AZD0156 in combination with irinotecan and 5FU was evaluated in preclinical models of CRC to determine whether low doses of AZD0156 enhance the cytotoxicity of irinotecan in chemotherapy regimens used in the clinic.

**Methods:**

Anti-proliferative effects of single-agent AZD0156, the active metabolite of irinotecan (SN38), and combination therapy were evaluated in 12 CRC cell lines. Additional assessment with clonogenic assay, cell cycle analysis, and immunoblotting were performed in 4 selected cell lines. Four colorectal cancer patient derived xenograft (PDX) models were treated with AZD0156, irinotecan, or 5FU alone and in combination for assessment of tumor growth inhibition (TGI). Immunofluorescence was performed on tumor tissues. The DDR mutation profile was compared across *in vitro* and *in vivo* models.

**Results:**

Enhanced effects on cellular proliferation and regrowth were observed with the combination of AZD0156 and SN38 in select models. In cell cycle analysis of these models, increased G2/M arrest was observed with combination treatment over either single agent. Immunoblotting results suggest an increase in DDR associated with irinotecan therapy, with a reduced effect noted when combined with AZD0156, which is more pronounced in some models. Increased TGI was observed with the combination of AZD0156 and irinotecan as compared to single-agent therapy in some PDX models. The DDR mutation profile was variable across models.

**Conclusions:**

AZD0156 and irinotecan provide a rational and active combination in preclinical colorectal cancer models. Variability across in vivo and in vitro results may be related to the variable DDR mutation profiles of the models evaluated. Further understanding of the implications of individual DDR mutation profiles may help better identify patients more likely to benefit from treatment with the combination of AZD0156 and irinotecan in the clinical setting.

**Supplementary Information:**

The online version contains supplementary material available at 10.1186/s12885-022-10084-7.

## Background

Drugs targeting DNA damage response (DDR) pathways are of great interest in oncology drug development. This interest stems from the ability of these drugs to target the DDR induced by cytotoxic chemotherapy and radiation therapy, which may serve as a mechanism of resistance to these treatments. The addition of a DDR inhibitor to a standard cytotoxic regimen thus provides a rational approach to improve standard therapies already known to benefit patients [1]. Such agents are also of specific interest in cancers that harbor defects in DDR pathway components, where they may create a synthetic lethal-like interaction, as in the case of poly (ADP-ribose) polymerase (PARP) inhibitors in BRCA1 or BRCA2 mutant cancers [2].

Ataxia-telangiectasia mutated (ATM) targeted agents are among the DDR inhibitors currently undergoing evaluation for cancer treatment. ATM is a serine threonine kinase that plays a key role in DNA double-strand break repair through both the homologous recombination repair and nonhomologous end joining pathways. ATM interacts with multiple other DDR factors, including the MRE11-NBS1-RAD50 (MRN) complex, as well as H2AX, MDM2, and CHK2 downstream [3].

Clinically available ATM inhibitors are currently limited to few compounds, including AZD0156, a highly selective and orally bioavailable ATM inhibitor with a cell IC_50_ value of 0.00058 μM. The IC_50_ values of related targets further demonstrate this selectivity: ATR 6.2 μM (cell), mTOR 0.61 μM (cell), PIK3Ca 1.4 μM (cell), DNAPK 0.14 μM (enzyme). Though no single-agent effect of AZD0156 was observed in an SW620 colorectal cancer xenograft mouse model, tumor regression was observed when combined with irinotecan. Combination effects were also observed with the addition of AZD0156 to olaparib in HBCx-10 patient derived triple-negative breast cancer (TNBC) xenografts harboring BRCA2 and TP53 mutations [4]. In additional TNBC models, AZD0156 was observed to enhance the effect of olaparib, even in models without DDR alterations. Furthermore, AZD0156 sensitized a large panel of cell lines including TNBC, gastric, and non-small cell lung cancer to olaparib through increasing DNA strand breaks which ultimately led to increased cell death [5].

Irinotecan is a topoisomerase 1 inhibitor that is used for the treatment of a variety of cancers, including colorectal cancer. A derivative of camptothecin, irinotecan leads to accumulation of reversible DNA single strand breaks that are converted into permanent single and double-strand breaks at the replication fork [6, 7]. Clinically, irinotecan is often used in combination with antimetabolite 5-fluorouracil (5FU) as FOLFIRI for the treatment of colorectal cancer.

The aim of this work is to evaluate AZD0156 in combination with irinotecan and 5FU in preclinical models of CRC to determine whether low doses of AZD0156 enhance the cytotoxicity of irinotecan in chemotherapy regimens used in the clinic.

## Methods

### Cell culture and reagents

Human CRC cell lines HCT8, RKO, LOVO, HT29, LS180, LS513, GPD2, LS1034, HCT116, DLD1, SW48, and Colo678 were obtained from American Type Culture Collection or Sigma, and identities were confirmed by DNA profiling at the Barbara Davis Molecular Biology Core. Cells were cultured in RPMI media supplemented with 10% FBS (Atlas Biologicals), 1% nonessential amino acids (Corning), and 1% penicillin/streptomycin. Cells were maintained in an incubator at 37° Celsius in 5% CO2 and routinely screened for the presence of mycoplasma (MycoAlert; Cambrex Bio Science).

AZD0156 was provided by AstraZeneca and was used for *in vitro* and *in vivo* experiments. The doses of AZD0156 selected for evaluation in this work were based on previously published data on the pharmacologic characteristics of the compound [4], as well as data evaluating AZD0156 at a dose of 30 nM in combination with olaparib [5]. Irinotecan was purchased from the University of Colorado Hospital Pharmacy and was used *in vivo*. The active metabolite of irinotecan, SN38, was purchased from Sigma and was used for *in vitro* experiments. 5FU was purchased from the University of Colorado Hospital Pharmacy and utilized in *in vitro* and *in vivo* experiments.

### Proliferation assays

Colorectal cancer cell lines were plated in 96-well plates and incubated overnight (1500–3000 cells per well based on growth characteristics). In order to calculate the single-agent IC_50_ of AZD0156, four CRC cell lines were exposed to increasing concentrations of AZD0156 for 72 h. Cell Titer Glo assay was used and IC_50_ was calculated using GraphPad Prism Software.

Cells from 12 CRC cell lines were exposed to DMSO, AZD0156 50 nM or 100 nM alone, SN38 10 nM alone, or the combination of SN38 and AZD0156 at each dose. Doses of SN38 and AZD-156 were selected based on prior work [8, 9]. Proliferation was assessed every 2–4 h over 120 h using the IncuCyte ZOOM™ live cell imaging system (Essen BioScience). Proliferation experiments were repeated × 3 for each cell line. The Bliss Additivity model was used to calculate synergy from average proliferation at 120 h [10, 11]. Values greater than 1 were considered consistent with a synergistic interaction between the agents evaluated. The Bliss Additivity model was selected over the Loewe method to assess synergy given AZD0156 does not reach EC_50_ in this work, and the Loewe method is based on the EC_50_ of each drug [12]. The use of the Bliss model here is also supported by its use in additional studies evaluating two agents with differing mechanisms of action, similar to this work [13–15].

### Clonogenic assays

Four of the CRC cell lines, HCT8, RKO, LOVO, and HT29, were seeded in 6-well plates (3000 cells per well) and incubated overnight to assess clonogenic colony formation [16, 17]. Cells were then exposed to vehicle, AZD0156 50 nM or 100 nM alone, SN38 2.5 nM alone, or the combination of SN38 and AZD0156 at each dose for 72 h. The higher dose of SN38 10 nM utilized in other in vitro work [9, 18] was associated with such minimal cellular confluence in SN38 treated plates that the lower 2.5 nM dose of SN38 was selected based on the dose titration for this assay. After 72 h the drug media was removed, and drug-free media was added for incubation until the vehicle well was confluent. Cells were next fixed with 100% methanol and stained with 1X crystal violet for 30 min, and then washed × 3 with water. Once dry, colony area was quantified using Image J (ColonyArea Plugin), as previously published [19]. Clonogenic assays were performed in triplicate, with representative images shown.

### Cell cycle analysis

HCT8, RKO, LOVO, and HT29 cells (300,000 per well) were seeded in 6-well plates containing 2 mL of complete media and incubated overnight. Cells were then exposed to vehicle, AZD0156 50 nM or 100 nM alone, SN38 10 nM alone, or the combination of SN38 and AZD0156 at each dose for 24 h. Next, the drug media was removed, and cells were trypsinized, washed with PBS, resuspended in Krishan’s stain, and incubated overnight at 4˚C. Cells were then analyzed by flow cytometry by the University of Colorado Cancer Center (UCCC) Flow Cytometry Core Facility. Cell cycle experiments were repeated three times for each cell line.

### Immunoblotting

HCT8, RKO, LOVO, and HT29 cells were seeded in 6-well plates and incubated overnight. Cells were then exposed to vehicle, AZD0156 50 nM or 100 nM alone, SN38 10 nM alone, or the combination of SN38 and AZD0156 at each dose for 24 and 72 h. Cells were then harvested with trypsin/EDTA and lysed in cell lysis buffer (Cell Signaling Technology) as previously described [20]. Electrophoresis of thirty micrograms of protein was performed on 4–12% Bis–Tris precast gels (Life Technologies, Carlsbad, CA) and then transferred to a nitrocellulose membrane using the Pierce G2 Fast Blotter (Pierce, Santa Ana, CA). Membranes were blocked at room temperature for 1 h in blocking buffer (0.1% Casein solution 0.2X PBS), and then exposed to blocking buffer with 0.1% Tween-20. The membranes were then incubated overnight at 4 °C with the following primary antibodies diluted 1:1000: P-RAD50 (Ser635), P-HH3 (Ser10), CHK2, P-CHK2, γH2AX (Ser139), and β-actin (Cell Signaling Technologies, Danvers, MA). Blots were washed in 1X TBS with 0.1% Tween-20 for 10 min × 3, and then incubated at room temperature for 1 h with the appropriate secondary goat anti-rabbit and goat anti-mouse immunoglobulin G (H + L) DyLight conjugated antibodies (Cell Signaling Technologies) at a 1:15,000 dilution. Blots were washed for 5 min × 3 with 1X TBST and then imaged using the Odyssey Infrared Imaging System (LI-COR, Lincoln, NE). Immunoblotting experiments were performed in triplicate, with representative images shown.

### Patient derived xenograft models

Patient derived xenograft models were generated as previously described [20]. Briefly, approximately 3 mm^3^ tumor sections were injected into the subcutaneous tissue of both flanks of immunodeficient female athymic nude (nu/nu) mice purchased from Envigo. (Indianapolis, IN). Tumor size was monitored twice weekly using digital calipers and recorded in the Study Director Program (Studylog, San Francisco, CA). Tumor volume was calculated using the following formula: (length × width^2^) × 0.52. When tumor volumes reached 150–200 mm^3^, mice were randomized to one of the following treatment groups: vehicle control, AZD0156 10 mg/kg by mouth (PO) 3 times weekly, irinotecan 15 mg/kg by intraperitoneal injection (IP) once weekly, 5FU 60 mg/kg IP once weekly, 5FU/irinotecan (CRC102 only), AZD0156/irinotecan, AZD0156/5FU, AZD0156/irinotecan/5FU. Dose selections were based on prior work [5, 21]. For randomization, mice were split into treatment groups, then tumor volumes were averaged within each group and hand adjusted to make group averages within 10 mm3 of each other. Following randomization, groups were assigned a treatment. There were at least six mice per treatment (*n* = 9–11 tumors per treatment). Tumors outside the indicated range were not used in measurement calculations, and mice with both tumors outside the range were excluded from treatment. Each treatment group was compared to the vehicle and the combination was compared to each single agent group using a 2-sided log-rank-test controlling for the type 1 error rate at 0.05. With 11 tumors per group, this allowed for 80.8% power to detect a median growth rate by end of study. For combination groups the chemotherapy was dosed followed by AZD0156 the next three days. All mice were housed on the same rack and were measured within the same hour, and dosed within the same hour when applicable. Tumor growth curves were plotted, and tumor growth inhibition values were calculated using the following formula: (TGI = [1-(average of final treated tumor volume/average of final vehicle tumor volume)] × 100). Specific growth rate was also assessed using GraphPad Prism 9.0. Mice were euthanized with cervical dislocation following anesthesia under isoflurane to ensure tumors were oxygenated. Animal studies were performed in a facility accredited by the American Association for Accreditation of Laboratory Animal Care in accordance with NIH guidelines and approved by the University of Colorado Institutional Animal Care and Use Committee prior to initiation (IACUC number 021).

### Immunofluorescence

Slides were fixed and blocked with 1% BSA and 1X PBS for 1 h in a humidified chamber at room temperature, then incubated overnight with γH2AX and P-RAD50 primary antibodies and washed with 1X PBS. Next slides were incubated with secondary antibodies AlexaFluor 488 and AlexaFluor 555 (Life Technologies) for 1 h in a humidified chamber at room temperature and washed in PBS. Counterstaining with DAPI in 1X PBS was performed for 30 min, followed by additional PBS wash. Slides were mounted with Fluoromount-G mounting media (SouthernBiotech), and imaged using Olympus FV-1000 confocal microscope at 20X magnification as described previously [22].

### Evaluation of dna damage repair mutations in *in vitro* and *in vivo* models

For the 12 cell lines evaluated, prototypical DDR mutations were evaluated based on data provided in the Cancer Cell Line Encyclopedia [23]. For evaluation of DDR profile of PDX models, whole exome sequencing was performed on tissues as described previously [24]. (Supplemental Table 1, Additional File 1).

### Statistical analysis

GraphPad Prism software version 9.0 was used to perform statistical analyses. One-way ANOVA with Tukey’s multiple comparisons test was used for all analyses. *P* = 0.05 was used as the cut-off for statistical significance. Error bars represent standard error of the mean.

## Results

### The combination of AZD0156 and SN38 affects proliferation and cellular regrowth in CRC cell lines

**Fig. 1:**
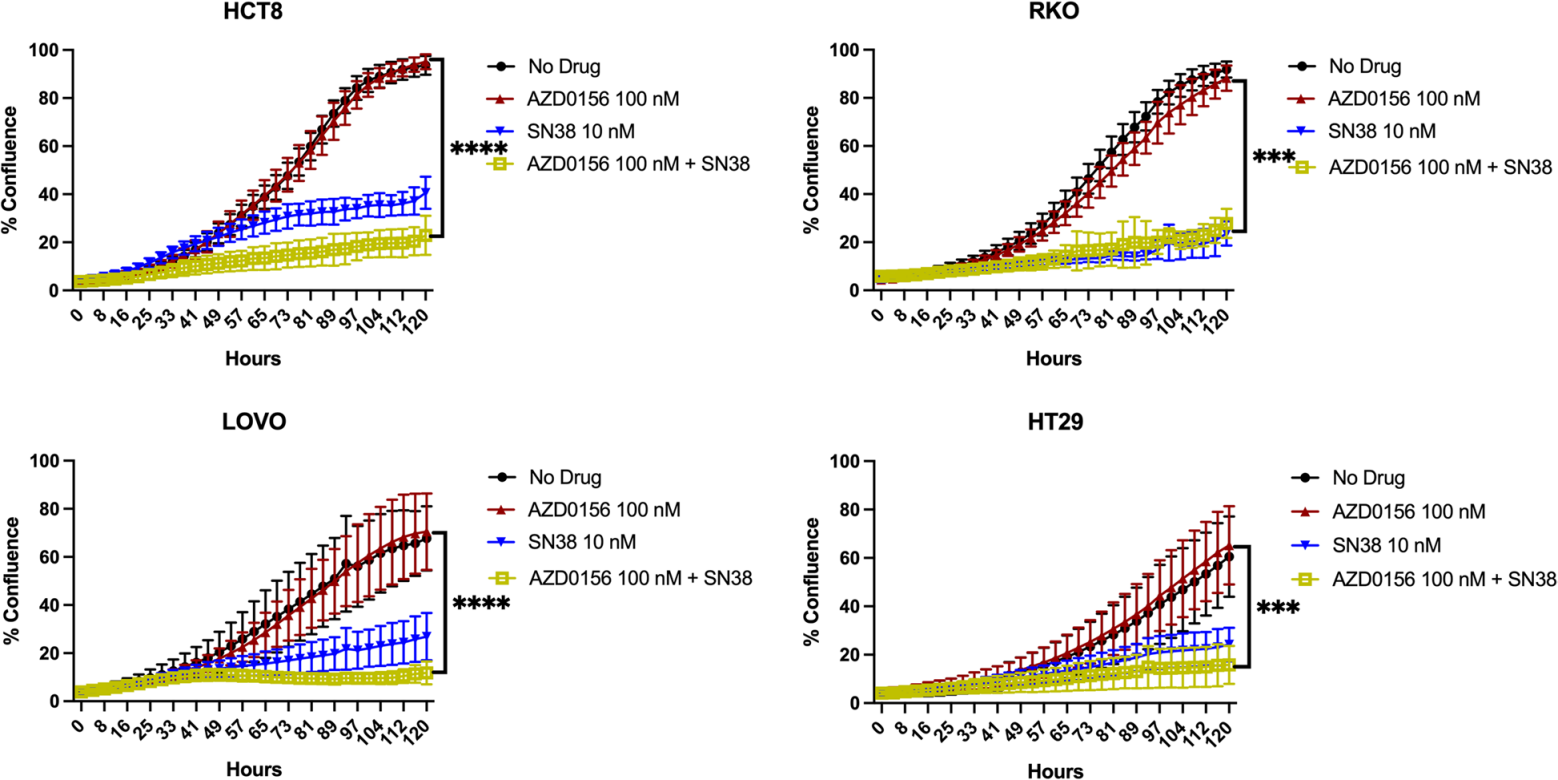
Effect of AZD0156 and SN38 on proliferation in CRC cell lines. Percent confluence relative to vehicle at 120 h as assessed by Incucyte ZOOM™. AZD0156 at a dose of 100 nM alone and in combination with SN38 at a dose of 10 nM. Experiments performed with 3 replicates. *** = *p* ≤ 0.001 and **** = *p* ≤ 0.0001 when comparing AZD0156 alone to combination with SN38

The IC_50_ across 4 CRC cell lines evaluated was close to 5 uM, though at these high doses there is concern for off-target effects of closely related kinases [4] (data not show). As anticipated, treatment with single-agent AZD0156 at a dose of 50 nM or 100 nM was not associated with a significant decrease in proliferation as compared to vehicle control across the 12 cell lines evaluated by IncuCyte assay (Fig. 1 and Supplemental Figs. 1 and 2, Additional File 1).

The four CRC cell lines with the best combination effect across the 12 tested (HCT8, RKO, LOVO, and HT29) were selected for further in vitro assessments. The combination of AZD0156 (100 nM) and SN38 (10 nM) resulted in decreased proliferation as compared to single-agent AZD0156 across all cell lines (HCT8 *P* < 0.0001, RKO *P* = 0.0005, LOVO *P* < 0.0001, HT29 *P* = 0.0002). When compared to single-agent SN38, the decrease in proliferation of the combination was not statistically significant, though a trend was noted in the HCT8 and LOVO cell lines (Fig. 1). This trend was also observed when the lower dose of AZD0156 (50 nM) was combined with SN38 (10 nM) (Supplemental Fig. 1, Additional File 1).

**Fig. 2:**
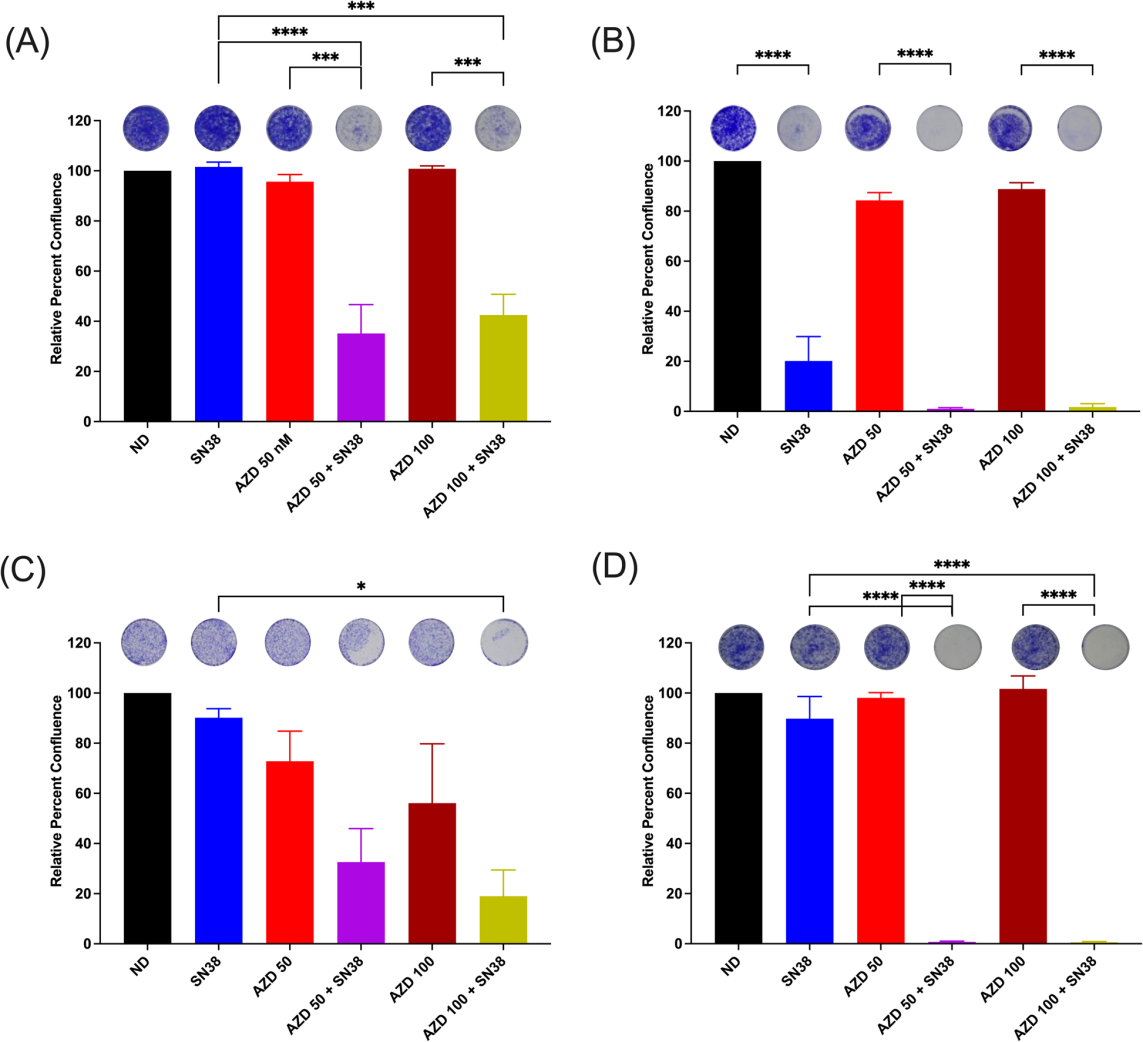
Effect of AZD0156 and SN38 on cellular regrowth in CRC cell lines. Percent confluence of CRC cells following exposure to SN38 2.5 nM and AZD0156 at 50 nM and 100 mM doses alone or in combination, followed by incubation in drug-free media for 72 h. **A** HCT8, **B** RKO, **C** LOVO, **D** HT29. Experiments performed with 3 replicates. * = *p* ≤ 0.05,*** = *p* ≤ 0.001,**** = *p* ≤ 0.0001. ND = No Drug; SN38 = SN38 2.5 nM; AZD 50 = AZD0156 50 nM; AZD 100 = AZD0156 100 nM

In the CRC cell lines, HCT8, RKO, LOVO and HT29, a decrease in percent confluence was observed by clonogenic assay following treatment with the combination of AZD0156 and SN38 followed by regrowth for 72 h. This decrease was statistically significant at the 50 nM dose of AZD0156 in combination with SN38 when compared to single-agent AZD0156 50 nM in the HCT8 (*P* = 0.0001), RKO (*P* < 0.0001) and HT29 (*P* < 0.0001) cell lines. There was a statistically significant decrease in percent confluence following treatment with this same combination when compared to SN38 in the HCT8 (*P* < 0.0001) and HT29 (*P* < 0.0001) cell lines. Similar patterns were seen at the 100 nM dose of AZD0156 in combination with SN38 when compared to single-agent AZD0156 (HCT8 *P* = 0.0002, RKO *P* < 0.0001, HT29 *P* < 0.0001), and single-agent SN38 (HCT8 *P* = 0.0002, RKO *P* < 0.0001, LOVO *P* = 0.0204, HT29 *P* < 0.0001) (Fig. 2).

The combination of AZD0156 100 nM and SN38 demonstrated synergy according to the Bliss Additivity model in the HCT8, LOVO, a HT29 cell lines, with values of 1.20, 1.38, and 1.68, respectively. A value of 1.04 was documented in the RKO model consistent with an additive interaction. When statistical analysis was performed as per Demidenko E, et al. [12], none of the 4 cell lines demonstrated statistically significant synergy (HCT8 *P* = 0.1213, RKO *P* = 0.9849, LOVO *P* = 0.0715, HT29 *P* = 0.6363), with findings more consistent with an additive effect of the AZD0156 and SN38 combination (Supplemental Fig. 3, Additional File 1).

### Increased G2/M arrest is observed in CRC cells upon exposure to the combination of AZD0156 and SN38 in CRC cell lines

**Fig. 3:**
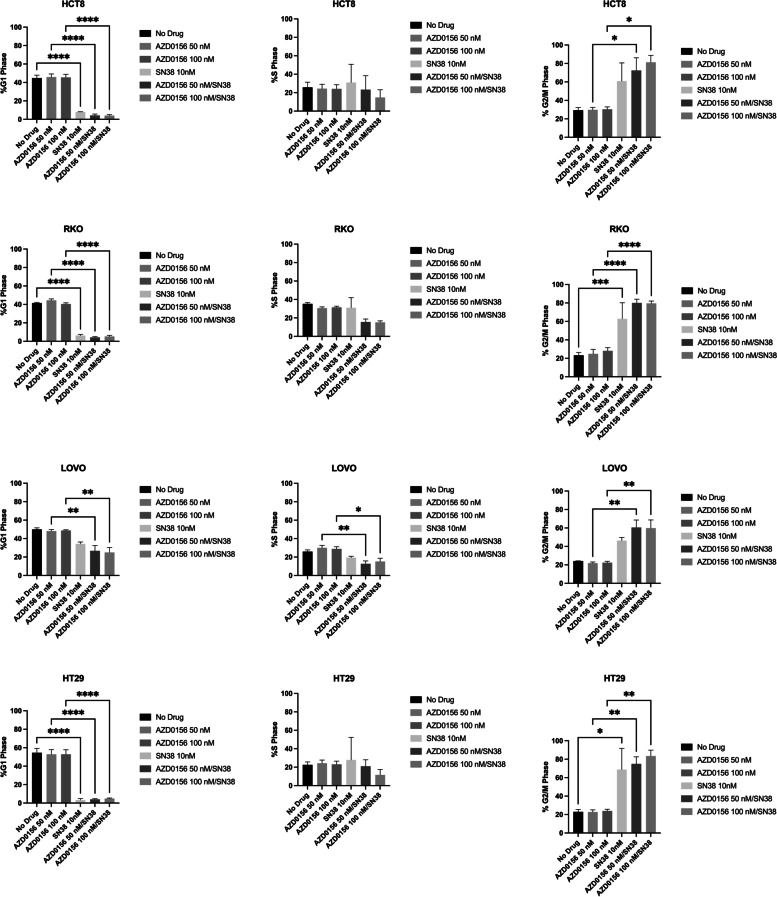
Effect of AZD0156 and SN38 on cell cycle progression in CRC cell lines. Cell cycle analysis by flow cytometry in CRC cells treated with AZD0156 50 nM or 100 nM and SN38 10 nM alone or in combination at 24 h. Data are presented as % cells in G1 Phase, S Phase, and G2/M Phase for each cell line evaluated. Experiments performed with 3 replicates. * = *p* ≤ 0.05, ** = *p* ≤ 0.01, *** = *p* ≤ 0.001, **** = *p* ≤ 0.0001

In the CRC cell lines, significant effects on the cell cycle were not observed upon treatment with AZD0156 as a single-agent. Significant decreases in G1 phase cells were noted in three of four cell lines exposed to SN38, as compared to untreated HCT8, RKO, and HT29 cells (*P* < 0.0001 for each). This corresponded to increased G2/M arrest, with statistically significant differences observed in the RKO (*P* = 0.0005) and HT29 (*P* = 0.0192) cell lines. When AZD0156 was added to SN38, further decreases in G1 phase cells were observed in cell lines with AZD0156 at 50 nM (HCT8 *P* < 0.0001, RKO *P* < 0.0001, LOVO *P* = 0.0089, HT29 *P* < 0.0001) and 100 nM (HCT8 *P* < 0.0001, RKO *P* < 0.0001, LOVO *P* = 0.0039, HT29 *P* < 0.0001) when compared to AZD0156 at respective doses as a single agent. The proportion of cells in G2/M phase increased accordingly with the combination of SN38 and AZD0156 at 50 nM (HCT8 *P* = 0.0384, RKO *P* < 0.0001, LOVO *P* = 0.0020, HT29* P* = 0.0035) and 100 nM (HCT8 *P* = 0.0129, RKO *P* < 0.0001, LOVO *P* = 0.0025, HT29* P* = 0.0012) (Fig. 3).

### Effectors of DNA Damage response are activated upon treatment with SN38 and decreased upon combination with AZD0156 in CRC cell lines

**Fig. 4:**
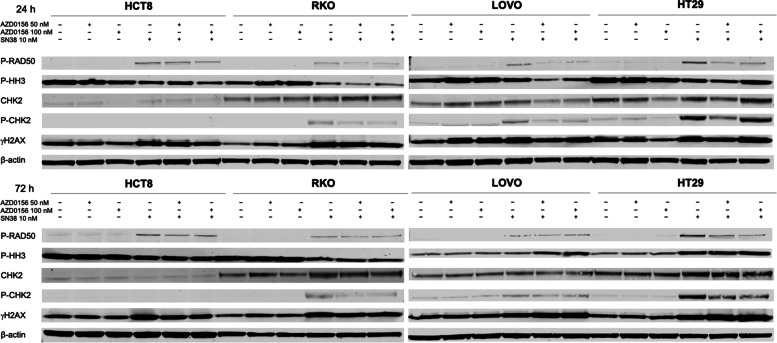
Effect of AZD0156 and SN38 on effectors of DDR in CRC cell lines. Immunoblots showing effects of AZD0156 at doses of 50 nM and 100 nM and SN38 10 nM alone and in combination on effectors of DDR and ATM activity at 24 and 72 h. Experiments performed with 3 replicates

P-RAD50 expression increased upon exposure to SN38 as compared to vehicle and single-agent AZD0156 treated samples across cell lines and at both 24 and 72 h, indicating induction of DNA damage repair pathways. A modest decrease in P-RAD50 expression was observed with the addition of AZD0156 to SN38 as compared to SN38 alone, indicating suppression of ATM signaling. This finding was most pronounced at the 24 h time point. Similar findings were observed with P-CHK2, a down-stream effector of ATM, with increase upon SN38 exposure and decrease in combination with AZD0156, in the majority of models, though in the HT29 model, P-CHK2 increased with the AZD0156 dose of 100 nM at the 24 h time point. In addition, the CHK2 protein was not detected in HCT-8 cells, as previously described [25]. γH2AX, a marker of DNA double-strand breaks, increased in cells exposed to SN38, and this was preserved with the addition of AZD0156. An increase in P-HH3 as a marker of G2/M arrest was also observed upon exposure to SN38 alone and in combination with AZD0156 in the LOVO and HT29 cell lines at 72 h (Fig. 4).

### The addition of AZD0156 to irinotecan-based chemotherapy leads to tumor growth inhibition in CRC PDX models

**Fig. 5:**
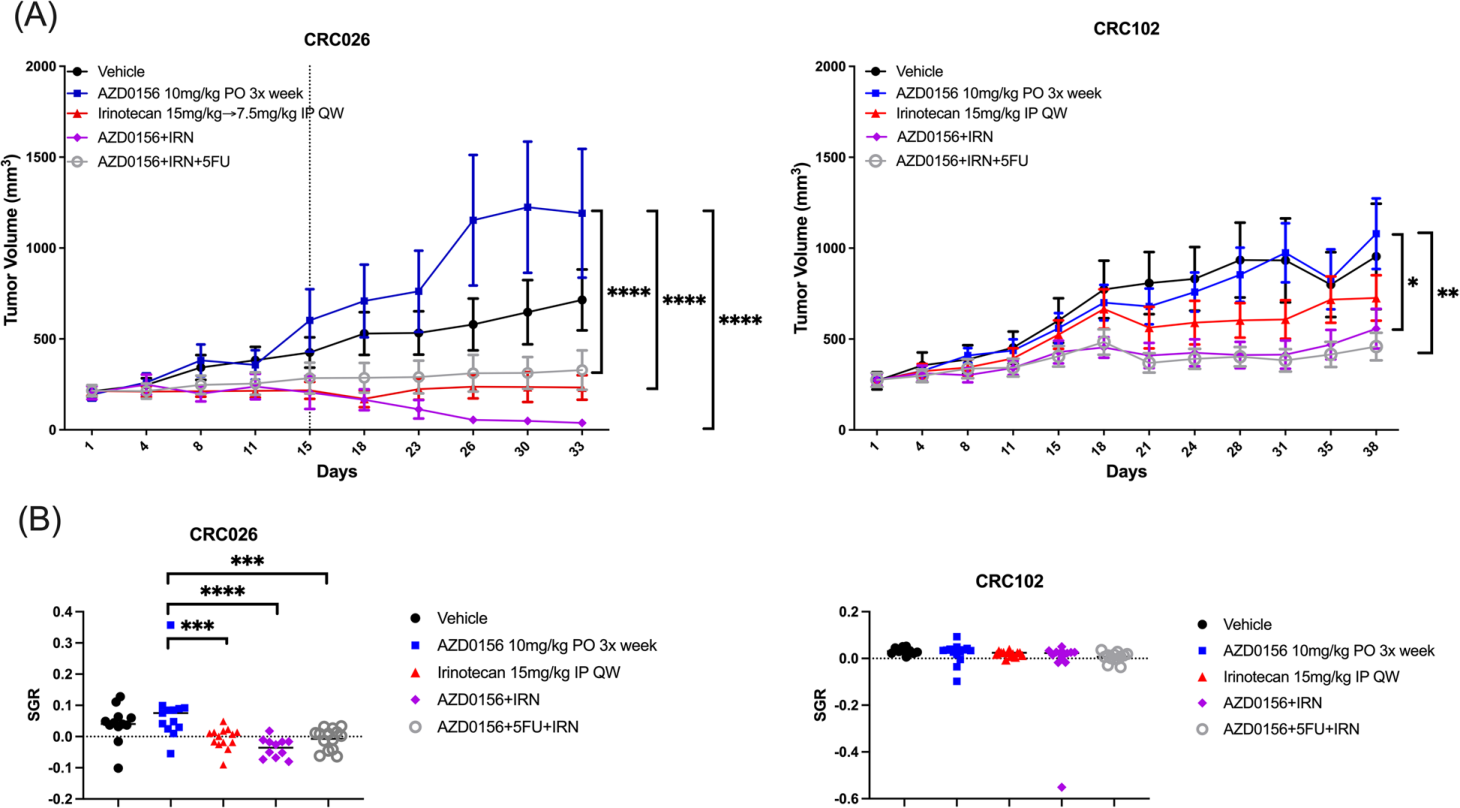
*In vivo* effects of AZD0156 and chemotherapy in CRC PDX models. **A** Effect of AZD0156 and irinotecan, alone and in combination with each other and 5FU in the CRC026 and CRC102 PDX models. Doses of irinotecan and 5FU were reduced in single-agent and combination arms at day 11 in the CRC026 model due to its sensitivity to these single agents. * = degree of statistical significance of treatment as compared to single agent AZD0156; * = *p* ≤ 0.05, ** = *p* ≤ 0.01, **** = *p* ≤ 0.0001. **B** Specific Growth Rates of CRC026 and CRC102; *** = *p* ≤ 0.001, **** = *p* ≤ 0.0001

In the CRC026 PDX model (NRAS Q61K MT), doses of irinotecan and 5FU were decreased from 15 mg/kg to 7.5 mg/kg and 60 mg/kg to 30 mg/kg, respectively, on day 11 of treatment due to the sensitivity of the model to these single-agents. Following these dose adjustments, a clear effect of the combination of AZD0156 and irinotecan as compared to single-agent AZD0156 (*P* < 0.0001) was observed, with evidence of tumor regression. Both single-agent irinotecan and the triplet of AZD0156, irinotecan, and 5FU were associated with significant tumor growth inhibition as well (*P* < 0.0001 for both). Interestingly, the addition of 5FU to the combination did not further enhance this effect, though tumor growth inhibition was not statistically significantly greater in the AZD0156/irinotecan arm as compared to the triplet arm. A trend toward tumor growth reduction was noted when comparing the combination of AZD0156 and irinotecan to single-agent irinotecan, though the difference was not statistically significant. Also of note in this model, a non-statistically significant increase in tumor volume was noted in the single-agent AZD0156 arm as compared to vehicle control (*P* = 0.1021). This pattern was not observed across other PDX models (Fig. 5A). No significiant findings were noted in additional treatment arms evaluated (Supplemental Fig. 4, Additional File 1). Tumor growth inhibition in the AZD0156/irinotecan combination arm was 94.7% as compared to 54.0% in the AZD0156/irinotecan/5FU triple combination arm.

In the CRC102 model (KRAS G12V MT), the AZD0156/irinotecan doublet (*P* = 0.0203) and AZD0156/irinotecan/5FU triple combination *(P* = 0.0090) were again associated with a statistically significant reduction in tumor volume when compared to single-agent AZD0156. A trend toward tumor growth reduction was noted in the AZD0156/irinotecan doublet (*P* = 0.0358) and AZD0156/irinotecan/5FU triplet as compared to single-agent irinotecan, but this was not statistically significant (Fig. 5A). No significiant findings were noted in additional treatment arms evaluated (Supplemental Fig. 4, Additional File 1). Tumor growth inhibition in the AZD0156/irinotecan/5FU triple combination arm was 51.1%, as compared to 43.5% in the AZD0156/irinotecan combination arm in this model.

Specific growth rate (SGR) analysis was performed for both models [26] (Fig. 5B). In the CRC026 model, a significant difference in specific growth rate was observed between the single-agent AZD0156 and the AZD0156/irinotecan doublet arm (*P* < 0.0001), as well as the triple combination arm (*P* = 0.0005) and single-agent irinotecan arm (*P* = 0.0007). When the doublet and triplet combination were compared to single-agent irinotecan, the differences were not significant. In the CRC102 model, no statistically significant differences in specific growth rate were noted.

In two additional PDX models, CRC001 (KRAS G12D MT) and CRC042 (KRAS G13D MT), less pronounced effects on tumor growth inhibiton were observed with the addition of AZD0156 to chemotherapy (Supplemental Fig. 5, Additional File 1). However, in the CRC042 model, the specific growth rate of the AZD0156/irinotecan doublet arm was lower than both the AZD0156 and single-agent irinotecan arms, and the SGR of triple combination arm was similarly lower than that of both the AZD0156 arm and irinotecan arm (Supplemental Fig. 5, Additional File 1).

### DNA damage response is affected by chemotherapy alone and in combination with AZD0156 in CRC PDX Models

**Fig. 6:**
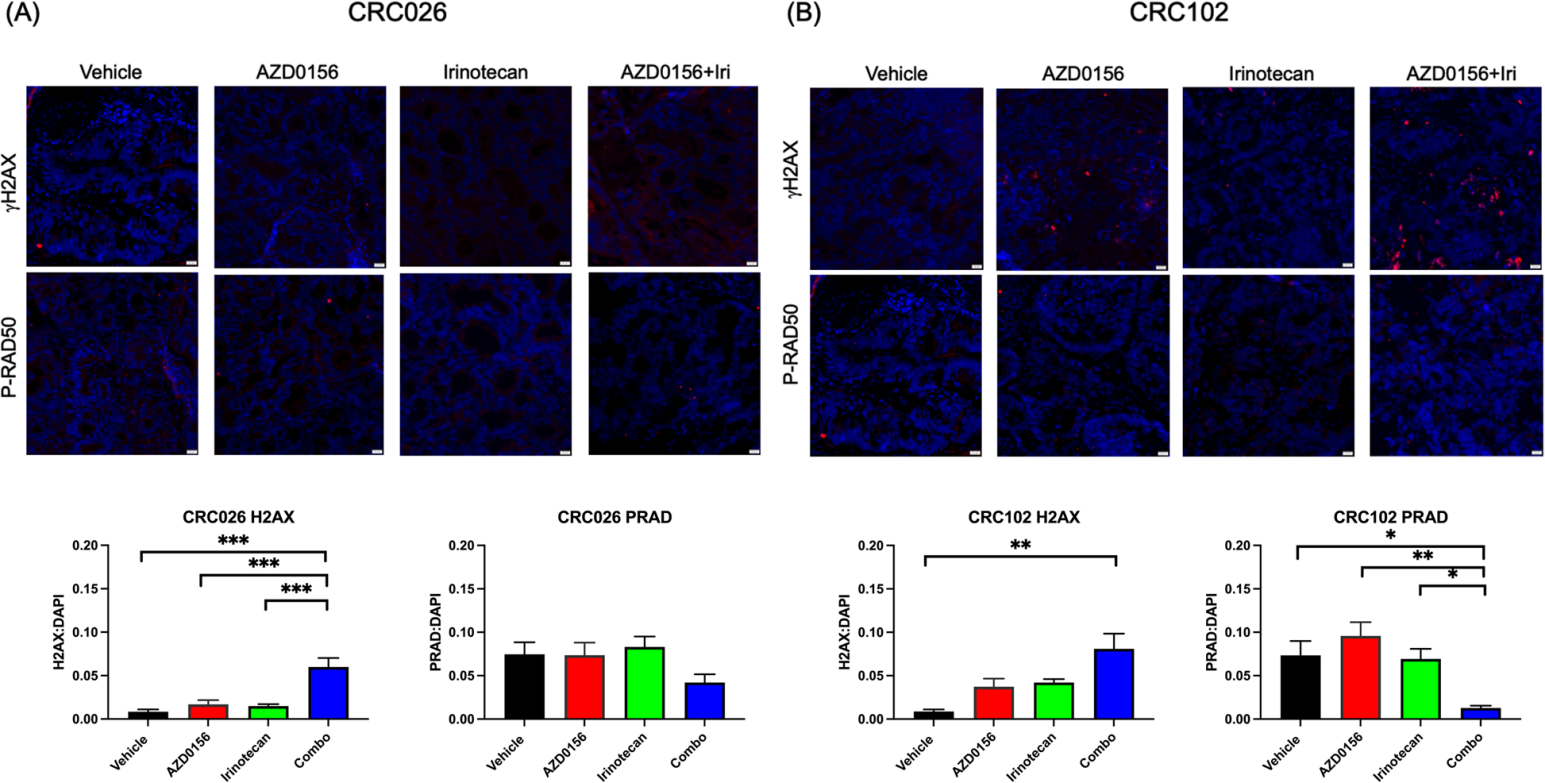
Effect of AZD0156 and SN38 on DNA damage repair in CRC PDX models. Immunofluorescence analysis of P-RAD50 and γH2AX as markers of DDR and ATM activation in CRC026 (**A**) and CRC102 (**B**) PDX tumor tissues at end of treatment. DAPI is seen in blue, γH2AX and PRAD-50, respectively, in red. Representative images at 20X magnification. Ratios of γH2AX and P-RAD50 to DAPI as quantified by immunofluorescence in each model; * = *p* ≤ 0.05, ** = *p* ≤ 0.01, *** = *p* ≤ 0.001

The pharmacodynamic effects of AZD0156 and irinotecan in CRC026 and CRC102 PDX models were evaluated by immunofluorescence assessment of γH2AX and P-RAD50 in tumor tissues at the end of treatment [27]. In the CRC026 model, this corresponds to 4 days following the last dose of irinotecan and 5FU, and 1 h following the last dose of AZD0156. In the CRC102 model, this corresponds to 6 days following the last dose of irinotecan and 5FU, and 1 h following the last dose of AZD0156. In the CRC026 model, a significant increase in γH2AX was observed in tumors from mice treated with the combination of irinotecan and AZD0156 as compared to single-agent AZD0156 (*P* = 0.0009) or irinotecan (*P* = 0.0005), consistent with increased accumulation of double-strand breaks in the setting of ATM inhibition (Fig. 6A). A similar trend was seen in CRC102 model, though the difference was only statistically significant when the combination arm was compared to control (Fig. 6B). In both models, P-RAD50 expression was decreased in tumors from mice treated with AZD0156 in combination with irinotecan, consistent with a decrease in DDR pathway activation. This difference was not significant in the CRC026 model, but was when compared to both AZD0156 (*P* = 0.0010) or irinotecan (*P* = 0.0265) as single-agents in the CRC102 model (Fig. 6).

### Alterations in prototypical DNA damage repair genes are variable across *in vivo* and *in vitro* models evaluated

Given the potential for improved response to ATM-targeted therapy in the setting of an underlying DDR alteration, prototypical DDR mutations were evaluated across all models utilized in this work (Supplemental Table 1, Additional File 1). All models evaluated had at least one mutation in a DDR gene, but there was no consistent mutation pattern across models. The HCT116 cell line was the only model evaluated that harbors an ATM mutation and was the only cell line evaluated in initial proliferation studies in which an antiproliferative effect was observed with AZD0156 as a single-agent. This finding was limited to the 100 nM dose. Interestingly, the cell lines in which the antiproliferative effect of SN38 was most enhanced by AZD0156, HCT8 and LOVO, both harbor CHEK2 mutations. The HT29 cell line, which had less pronounced, though still statistically significant enhancement of antiproliferative chemotherapy effects with AZD0156, has an TP53 mutation, with no other DDR alterations.

Though the DDR mutation profile of the PDX models evaluated was overall similar, it is possible that key variations impacted treatment effect. In the CRC026 model, in which the most pronounced tumor growth inhibition was observed with AZD0156 and irinotecan, the only unique alteration identified was an NRAS Q61K mutation. The CRC102 model harbors a TP53 mutation, which may explain the altered anti-tumor activity associated with single-agent chemotherapy, and improved anti-tumor activity with the AZD0156/irinotecan/5FU triplet. Interestingly, the CRC042 model, in which the tumor specific growth rate was significantly decreased with the addition of AZD0156 to irinotecan, does harbor an ATR mutation.

## Discussion

This data indicates that single-agent AZD0156 has limited activity in colorectal cancer models, but when combined with a cytotoxic chemotherapeutic agent that induces DNA double-strand breaks (irinotecan or SN38), is associated with greater effect in both CRC cell lines as well as in PDX models. Though a trend toward enhancement in the cytotoxicity of SN38 and irinotecan was observed with the addition of low doses of AZD0156 in these models, these trends were not statistically significant.

SN38 has been previously shown to induce a G2/M cell cycle arrest [28], which was further confirmed in our data, with significant increase in cells in G2/M in two cell lines, accompanied by a corresponding decrease in the G1 fraction. These findings were maintained when SN38 was combined with AZD0156 at both doses, with a statistically significant increase in G2/M arrest noted in all 4 cell lines. Further confirming these results were findings of increased phosphorylated CHK2 by immunoblotting, which has been associated with G2/M arrest [29]. These changes were observed in the RKO, LOVO, and HT29 cell lines at both doses at 24 and 72 h, with the exception of the 100 nM dose of AZD0156 combined with SN38 in the HT29 cell line at 24 h. This is not observed in the cell cycle analysis of the AZD0156 100 nM combination in HT29 cells at 24 h, where increase in G2/M fraction is significantly increased as compared to single-agent AZD0156. In addition, the lack of CHK2 protein in HCT-8 cells has been previously described [25], and was not attributed to altered cell cycle arrest in these cells. Similar increases in P-HH3 by immunoblotting, which has been observed to increase in G2/M arrest [30], were seen only in the LOVO and HT29 cell lines at the 72 h time point, and not at the 24 h time point, though cell cycle analyses performed at 24 h indicate significant increase in G2/M arrest across all cell lines evaluated.

In addition to indicating cycle cycle arrest, these observed changes in P-CHK2 by immunoblotting suggest suppression of ATM signaling and DNA damage response induced by AZD0156. This is further supported by a similar pattern of P-CHK2 expression in prior work with the combination of AZD0156 and olaparib [5]. However, similar findings for the phospho-PRAD50 protein were not as conclusive, with less consistent decreases in the P-RAD50 proteins with addition of AZD0156 in the HCT8 and RKO cell lines, as well as the LOVO cell line at 72 h. A similar trend of P-RAD50 is observed in the CRC026 and CRC102 PDX tumor tissues at the end of treatment by immunofluorescence, though decreases were only significant in the CRC102 model. The pattern of increased γH2AX by immunoblotting across cell lines and time points, with the exception of the LOVO cell line at 24 h, demonstrates increased accumulation of DNA double-strand breaks in the setting of SN38 exposure. However, in immunofluorescence data from PDX models, γH2AX is further increased in the AZD0156/irinotecan treated models, suggesting further accumulation of DNA double-strand breaks, due to inhibition of ATM when combined with irinotecan, though this is only significant in the CRC026 model.

Though 5FU as an antimetabolite chemotherapeutic agent does not induce DNA double-strand breaks, given the frequent clinical use of the combination as FOLFIRI for the treatment of CRC this compound was tested in combination with AZD0156 and irinotecan *in vivo*. Though in most of the PDX models evaluated, the triplet combination of AZD0156/irinotecan/5FU was associated with similar tumor growth inhibition as the doublet combinations, the finding of tumor regression in the AZD0156/irinotecan treated arm of the CRC026 model is notable. This is also the model in which the doses of both irinotecan and 5FU were adjusted at day 15 due to significant single-agent sensitivity, and it is possible that both the sensitivity of the model and the adjustment could have impacted these findings. Overall, the addition of 5FU to the AZD0156/irinotecan combination is that it did not induce a negative effect on the doublet therapy. However, the lack of added benefit of this additional cytotoxic agent may provide an opportunity to minimize the toxicity of a dual-cytotoxic regimen for the treatment of CRC through substitution of a targeted agent.

Given the diverse DDR alterations noted across models evaluated in this work, it is possible that variability in both *in vitro* and *in vivo* results described here may be in part related to these variable molecular profiles. Prior work in other ATM inhibitors has evaluated this relationship, though without clear correlation. For example, in colorectal cancer cell lines, enhancement of the effect of radiation and topoisomerase I and II agents with ATM inhibitor KU59403 was not affected by p53 mutation status [31]. However, in orthotopic xenograft models of glioblastoma multiforme, increased sensitivity to the combination of ATM inhibitor KU-60019 combined with radiation was observed in p53-mutant models [32]. Improved understanding of the relationship between the various alterations in the DDR pathway and the effect of ATM inhibitors may allow for identification of predictive biomarkers which could be used to select patients most likely to benefit from ATM inhibitors in the clinic. It is possible that alterations in a variety of genes with roles in DDR pathway response may be associated with improved treatment effect of AZD0156 and other ATM inhbitors, and in addition to further exploration in the preclinical setting, evaluating for such alterations in clinical trials of ATM and other DDR pathway-targeting agents could help better identify potential biomarkers predictive of treatment response. In addition to further assessment of somatic mutations in the DDR pathway, there may also be benefit to evaluation of germline mutations, similar to the use of germline and somatic BRCA mutations to select patients for PARP inhibitor therapies in various clinical settings [33].

We have previously evaluated the combination of early-generation ATM inhibitor AZ31 with SN38 / irinotecan. In this work, a combination effect of AZ31 and SN38 is documented, and determined to be related to cell cycle arrest, rather than apoptosis [21]. In addition, prior work has shown AZD0156 as a single-agent to induce little apoptosis [5]. Based on these prior data, we believe the combination effect observed with AZD0156 and SN38 / irinotecan is related to cell cycle arrest, rather than apoptosis.

However, there are some differences in our data as compared to prior work with AZ31 and other ATM inhibitors. For example, an improved combination effect of AZ31 and irinotecan was observed in the setting of irinotecan resistance [21], while in this work, there was no clear link between irinotecan resistance and response to combination therapy. In prior work evaluating AZD0156, a more robust suppression of P-RAD50 and P-CHK2 is demonstrated with the addition of AZD0156 to radiation than is seen in this data, though difficult to interpret in the setting of a different source of DNA damage (radiation vs chemotherapy) and a different cancer cell type (FaDu squamous cell hypopharyngeal cancer cells) [5].

The key limitation of this work are the variations *in vivo* and *in vitro* results described above. Though we believe the varied DDR alterations present across the models evaluated in this work likely contribute to this variability, a clear relationship was not identified in this work, and will require further assessment in the future.

## Conclusions

Our results suggest that the addition of AZD0156 to irinotecan enhances the effect of AZD0156, which is otherwise limited in CRC models. However, despite suggestive trends, a significant enhancement in the effect of irinotecan was not demonstrated. Variability in results across *in vitro* and *in vivo* models indicate that the effect of the combination in colorectal cancer may be influenced by the underlying DDR phenotype of the individual tumor. Further understanding of this relationship in future studies will be important in identifying a subpopulation of colorectal cancer patients most likely to benefit from treatment with this combination.

## Supplementary Information

**Additional file 1: Supplemental Table 1. **Prototypical DNA Damage Repair Mutations in CRC Models Utilized in this Study. **Supplemental Figure 1.** Effect of AZD0156 and SN38 on proliferation in CRC cell lines. AZD0156 dose 50nM, SN38 dose 10nM. Percent confluence relative to vehicle at 120 hours as assessed by Incucyte ZOOM. **Supplemental Figure 2.** Effect of AZD0156 and SN38 on proliferation in additional CRC cell lines. Percent confluence relative to vehicle as measured by IncuCyte ZOOM™ over 120 hours in an additional 8 CRC cell lines treated with AZD0156 50 nM and 100 nM and SN38 10 nM, alone and in combination. **Supplemental Figure 3.** Statistical evaluation of synergy based on Bliss independence. Comparison of single agent and combination effect as assessed by Incucyte ZOOM™ to Bliss independence, derived from individual groups by computing survival fraction under the assumption that treatments act independently, as per Demidenko E, et al. [12]. **Supplemental Figure 4.** *In vivo* effects of AZD0156 and chemotherapy in CRC PDX models. (A) Effect of AZD0156, irinotecan, and 5FU, alone and in combination in the CRC026 and CRC102 PDX models. Additional treatment groups added to data provided in Figure 5 of the main text. Doses of irinotecan and 5FU were reduced in single-agent and combination arms at day 11 in the CRC026 model due to its sensitivity to these single agents. (B) Specific Growth Rates of CRC026 and CRC102. **Supplemental Figure 5.** (A) Effect of AZD0156, irinotecan, and 5FU, alone and in combination in additional CRC001 and CRC042 PDX models. (B) Specific growth rates of CRC001 and CRC042.

**Additional file 2. **Original, full-length images from immunoblotting, corresponding to cropped images shown in Fig. 4.

**Additional file 3. **ARRIVE guidelines checklist referencing details of animal experiments as outlined in the manuscript.

## Data Availability

All data that supports the findings of this study are available in this published article and the supplementary information files.

## Abbreviations

5FU: 5-Fluorouracil
ATM: Ataxia telangiectasia mutated
CRC: Colorectal cancer
DDR: DNA damage response
IACUC: Institutional Animal Care and Use Committee
PARP: Poly (ADP-ribose) polymerase
PDX: Patient derived xenograft
SGR: Specific growth rate
TNBC: Triple-negative breast cancer
TGI: Tumor growth inhibition
UCCC: University of Colorado Cancer Center
