# Supplementary material for: ATM kinase inhibitor AZD0156 in combination with irinotecan and 5-fluorouracil in preclinical models of colorectal cancer
Source: BMC Cancer. 2022 Oct 29;22:1107. doi: 10.1186/s12885-022-10084-7 (PMC9617348; doi:10.1186/s12885-022-10084-7)
Supplement: Supplementary file 1 — Additional file 1: Supplemental Table 1. Prototypical DNA Damage Repair Mutations in CRC Models Utilized in this Study. Supplemental Figure 1. Effect of AZD0156 and SN38 on proliferation in CRC cell lines. AZD0156 dose 50nM, SN38 dose 10nM. Percent confluence relative to vehicle at 120 hours as assessed by Incucyte ZOOM. Supplemental Figure 2. Effect of AZD0156 and SN38 on proliferation in additional CRC cell lines. Percent confluence relative to vehicle as measured by IncuCyte ZOOM™ over 120 hours in an additional 8 CRC cell lines treated with AZD0156 50 nM and 100 nM and SN38 10 nM, alone and in combination. Supplemental Figure 3. Statistical evaluation of synergy based on Bliss independence. Comparison of single agent and combination effect as assessed by Incucyte ZOOM™ to Bliss independence, derived from individual groups by computing survival fraction under the assumption that treatments act independently, as per Demidenko E, et al. [12]. Supplemental Figure 4. In vivo effects of AZD0156 and chemotherapy in CRC PDX models. (A) Effect of AZD0156, irinotecan, and 5FU, alone and in combination in the CRC026 and CRC102 PDX models. Additional treatment groups added to data provided in Figure 5 of the main text. Doses of irinotecan and 5FU were reduced in single-agent and combination arms at day 11 in the CRC026 model due to its sensitivity to these single agents. (B) Specific Growth Rates of CRC026 and CRC102. Supplemental Figure 5. (A) Effect of AZD0156, irinotecan, and 5FU, alone and in combination in additional CRC001 and CRC042 PDX models. (B) Specific growth rates of CRC001 and CRC042. [file 12885_2022_10084_MOESM1_ESM.pdf]

Supplemental Table 1: Prototypical DNA Damage Repair Mutations in CRC Models Utilized in this Study

	CRC001	CRC026	CRC042	CRC102	Colo678	DLD1	GP2D	HCT116	HCT8	HT29	LOVO	LS180	LS513	LS1034	RKO	SW48
ATM	WT	WT	WT	WT	WT	WT	WT	A1127V	WT	WT	WT	WT	WT	WT	WT	WT
ATR	WT	WT	R2112C	WT	WT	I1851V	P1605L	WT	WT	WT	WT	WT	WT	WT	WT	I2376V
ATRX	WT	WT	WT	WT	WT	WT	D852E	T1529del	R2474C	WT	WT	WT	WT	WT	WT	ET886fs
ATRIP	WT	WT	WT	WT	WT	WT	WT	WT	WT	WT	WT	WT	WT	WT	WT	L327fs
CHEK1	ND	WT	WT	WT	WT	WT	R419S	WT	WT	WT	WT	WT	WT	WT	WT	WT
CHEK2	WT	WT	WT	WT	WT	A247D	WT	L355P	A290D/ R188W	WT	P431fs	WT	WT	WT	WT	WT
BRCA1	WT	Q356R	L1850M	E1038G	WT	P1190H	WT	WT	E1038G	WT	WT	N810T	WT	WT	D435Y	WT
BRCA2	N2553D	WT	WT	WT	WT	Q1782fs	Q1452R/ R2973R	IK2672fs	N289H/ H2248L/ R2784Q	WT	WT	H962H/ T3030fs	WT	WT	Q1782fs	Q1782fs
TP53	WT	WT	WT	R249S	WT	S241F	WT	WT	WT	R273H	WT	WT	WT	G245S	WT	WT
FANCA	WT	WT	WT	WT	WT	WT	R49R	P615fs	L637M	WT	R350W	R1084C	WT	WT	E345fs	WT
FANCD2	WT	WT	WT	WT	WT	R1273Q	WT	WT	R1273Q	WT	WT	WT	WT	WT	WT	D1350N
CCNE1	WT	WT	WT	WT	WT	N260I	WT	WT	N260I	WT	WT	WT	WT	WT	WT	WT
RPA1	WT	WT	WT	WT	Q420K	WT	WT	WT	WT	WT	WT	WT	WT	WT	WT	WT
RPA2	WT	ND	WT	WT	WT	WT	WT	WT	WT	WT	WT	G204D	WT	WT	WT	WT
TOPBP1	WT	ND	WT	WT	WT	ND	WT	WT	R309C	WT	WT	WT	WT	WT	WT	F24fs
XRCC1	Q399R	Q399R	Q399R	Q399R	WT	WT	WT	WT	WT	WT	WT	WT	WT	WT	WT	WT
MRE11A	WT	WT	WT	WT	A47S	WT	A542A	WT	WT	WT	WT	WT	WT	WT	WT	WT
RAD50	WT	WT	WT	WT	WT	WT	WT	L719fs	WT	WT	L719fs	LK719fs	WT	WT	L719fs	WT
NRAS	WT	Q61K	WT	WT	WT	WT	WT	WT	WT	WT	WT	WT	WT	WT	WT	WT
KRAS	G12D	WT	G13D	G12V	G12D	G13D	G12D	G13D	G13D	WT	G13D	G12D	G12D	A146T	WT	WT
MYC	WT	WT	WT	WT	WT	WT	I129V	WT	WT	WT	WT	WT	WT	WT	A336V	WT

WT: Wild type; MT: Mutant; ND: Not detected. PDX mutation data (19). Cell line mutation data (18).

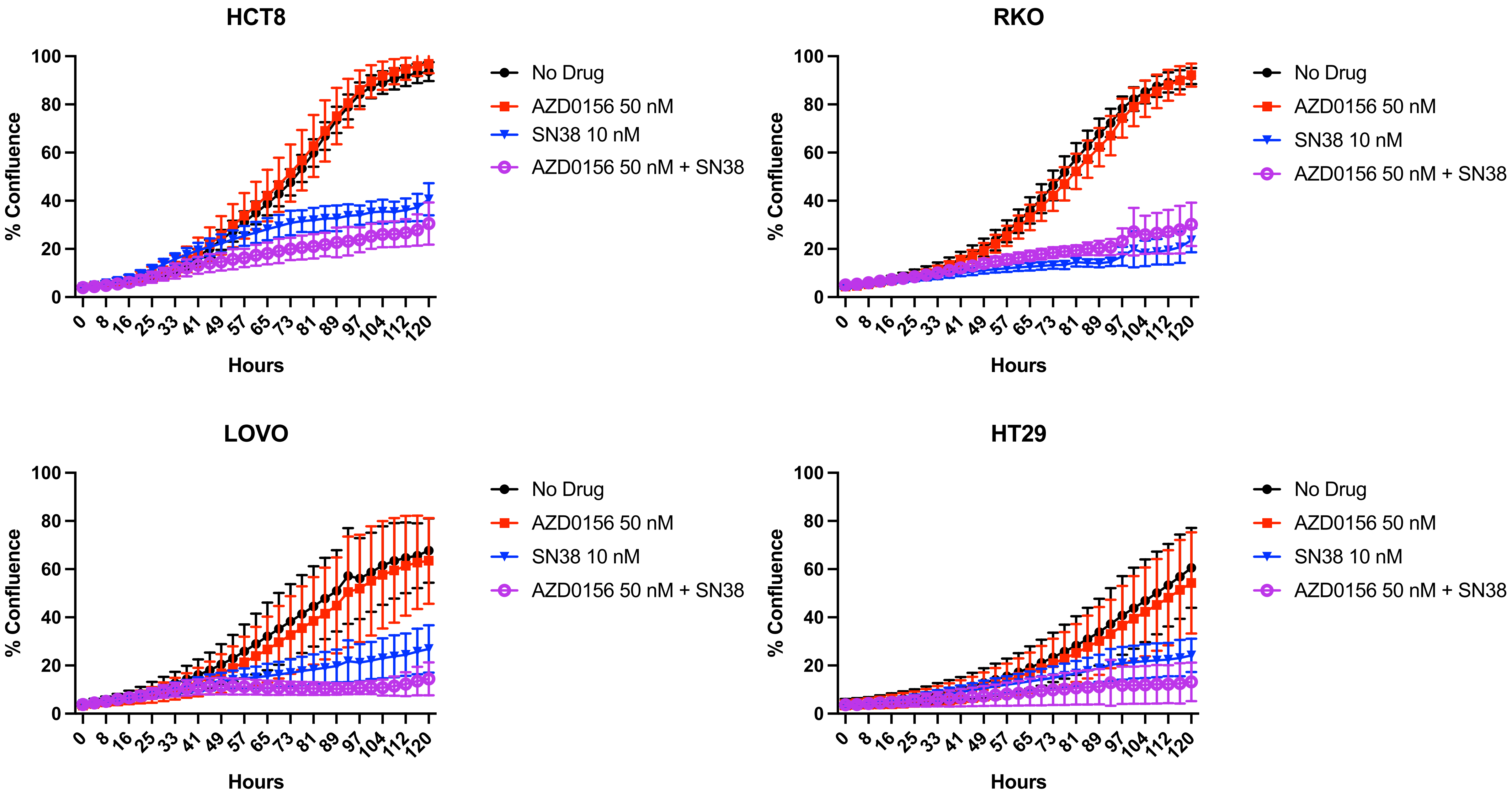

Supplemental Figure 1. Effect of AZD0156 and SN38 on proliferation in CRC cell lines. AZD0156 dose 50nM, SN38 dose 10nM. Percent confluence relative to vehicle at 120 hours as assessed by Incucyte ZOOM™

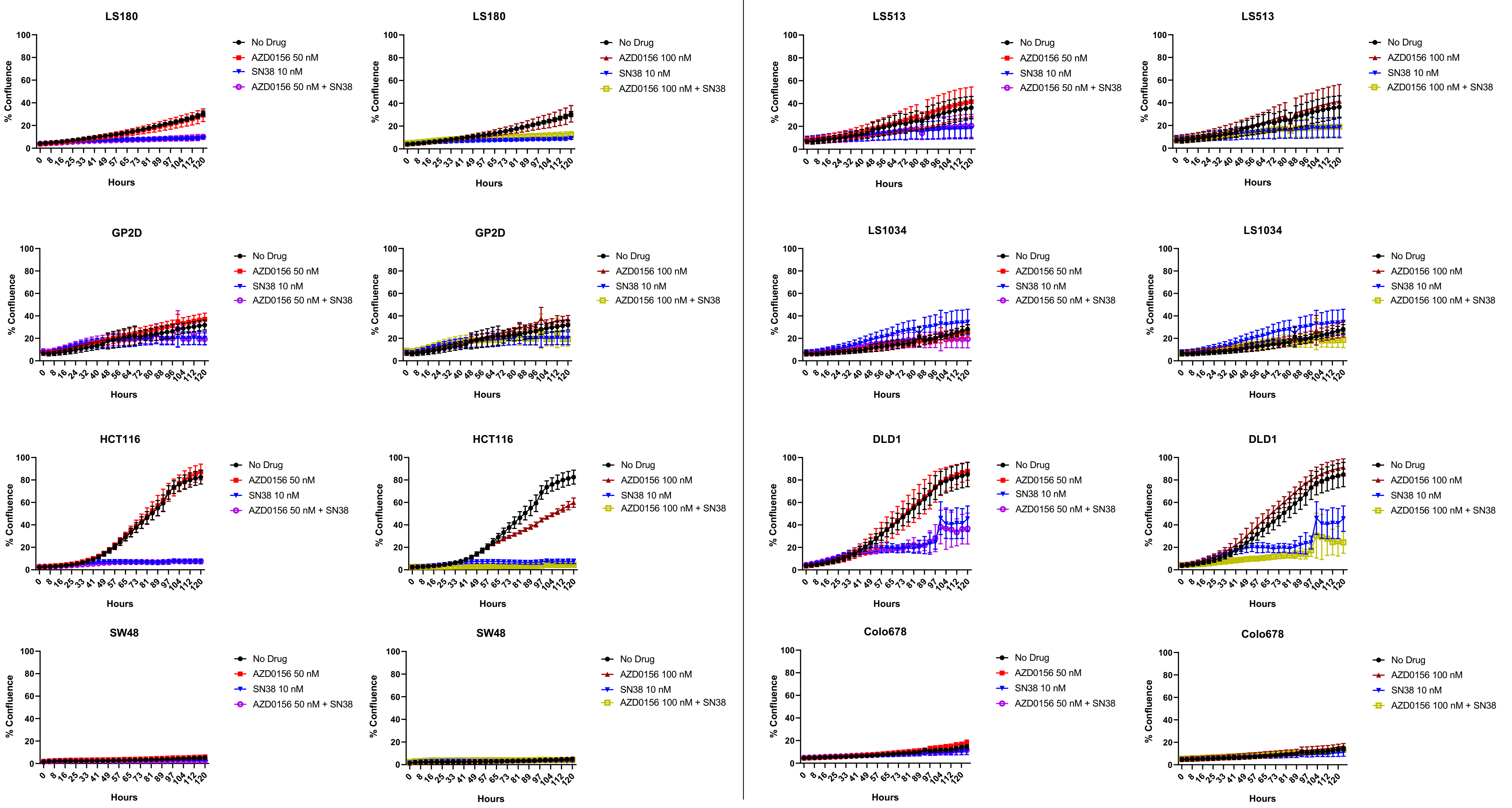

Supplemental Figure 2. Effect of AZD0156 and SN38 on proliferation in additional CRC cell lines. Percent confluence relative to vehicle as measured by IncuCyte ZOOM™ over 120 hours in an additional 8 CRC cell lines treated with AZD0156 50 nM and 100 nM and SN38 10 nM, alone and in combination.

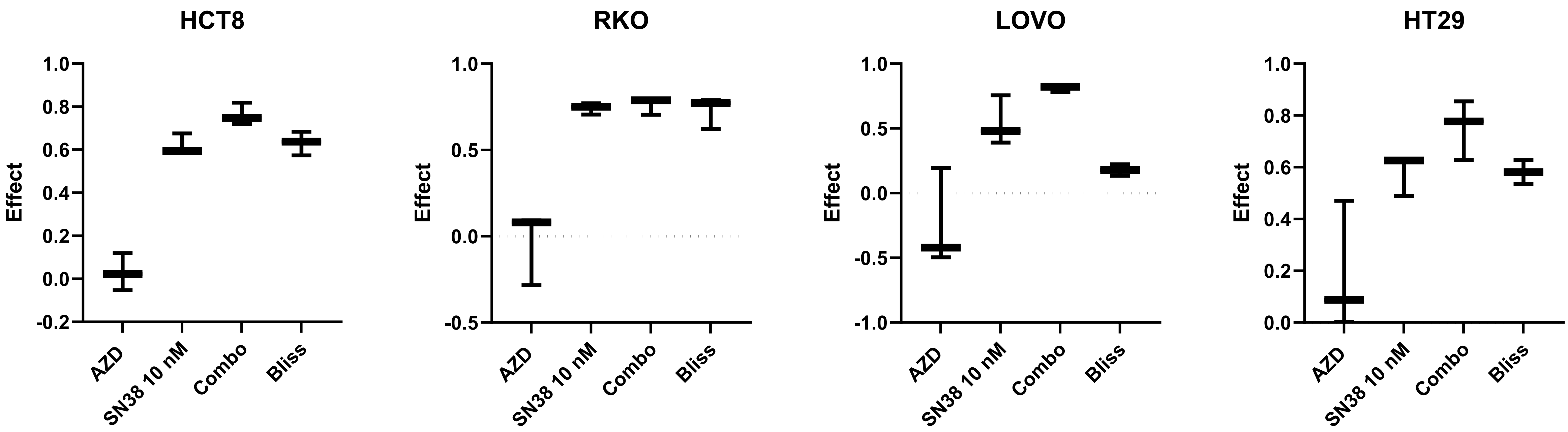

Supplemental Figure 3. Statistical evaluation of synergy based on Bliss independence. Comparison of single agent and combination effect as assessed by Incucyte ZOOM™ to Bliss independence, derived from individual groups by computing survival fraction under the assumption that treatments act independently, as per Demidenko E, et al. (21).

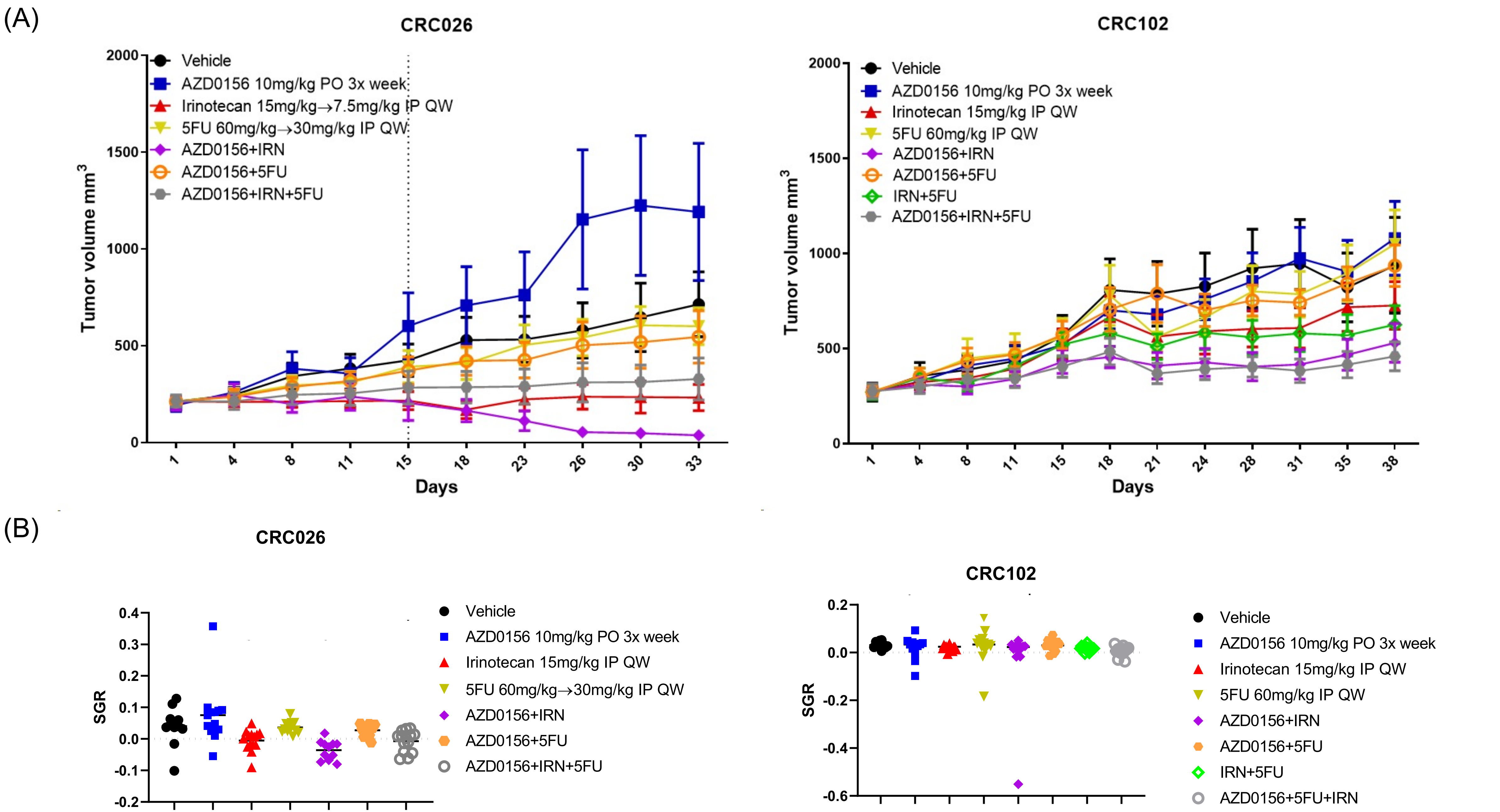

Supplemental Figure 4. ***In vivo*** **effects of AZD0156 and chemotherapy in CRC PDX models. (A)** Effect of AZD0156, irinotecan, and 5FU, alone and in combination in the CRC026 and CRC102 PDX models. Additional treatment groups added to data provided in Figure 5 of the main text. Doses of irinotecan and 5FU were reduced in single-agent and combination arms at day 11 in the CRC026 model due to its sensitivity to these single agents. **(B)** Specific Growth Rates of CRC026 and CRC102

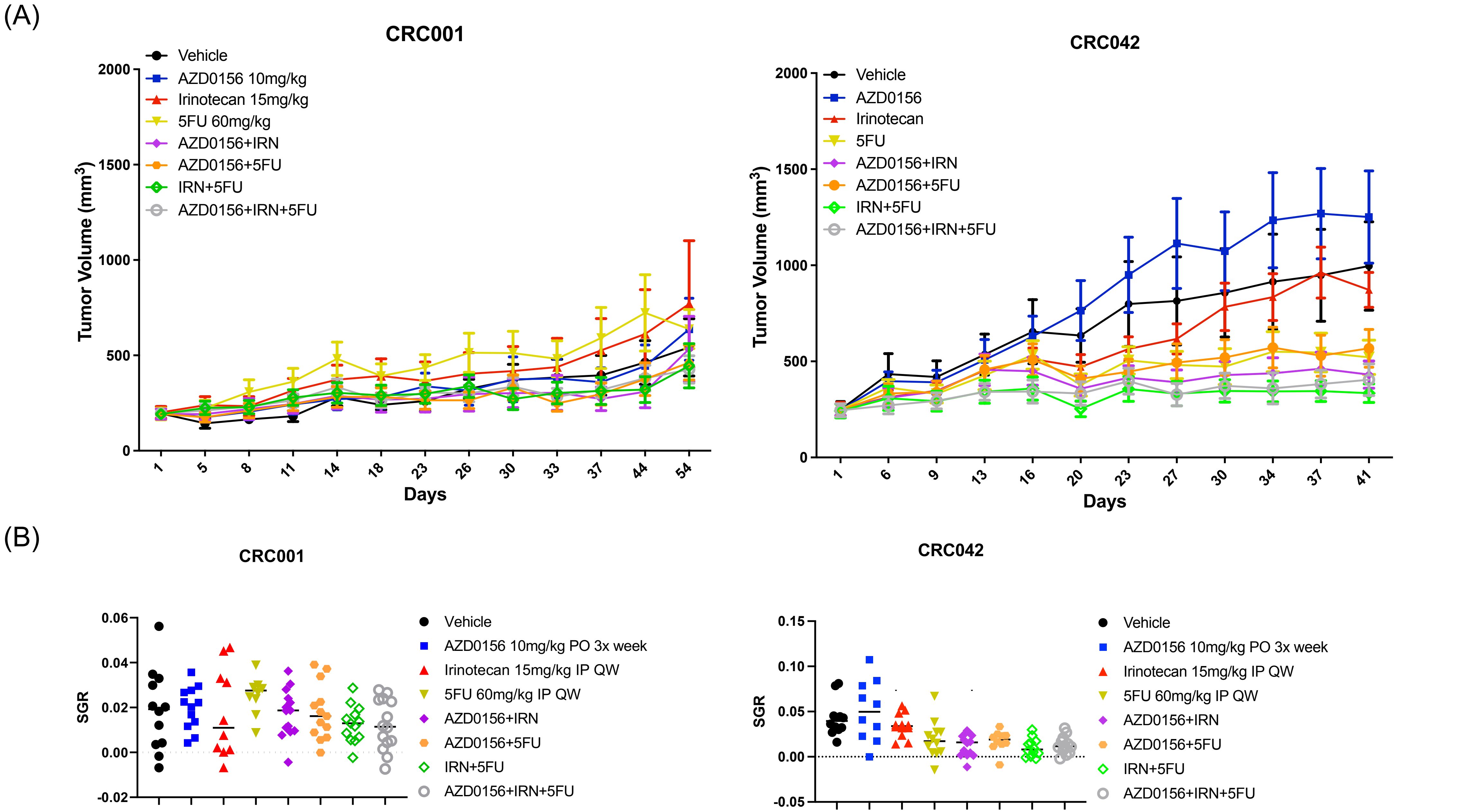

Supplemental Figure 5. ***In vivo*** **effects of AZD0156 and chemotherapy in additional CRC PDX models. (A)** Effect of AZD0156, irinotecan, and 5FU, alone and in combination in additional CRC001 and CRC042 PDX models. (B) Specific growth rates of CRC001 and CRC042
