# Supplementary material for: ATM kinase inhibitor AZD0156 in combination with irinotecan and 5-fluorouracil in preclinical models of colorectal cancer
Source: BMC Cancer. 2022 Oct 29;22:1107. doi: 10.1186/s12885-022-10084-7 (PMC9617348; doi:10.1186/s12885-022-10084-7)
Supplement: Supplementary file 2 — Additional file 2. Original, full-length images from immunoblotting, corresponding to cropped images shown in Fig. 4. [file 12885_2022_10084_MOESM2_ESM.pdf]

24 HOURS

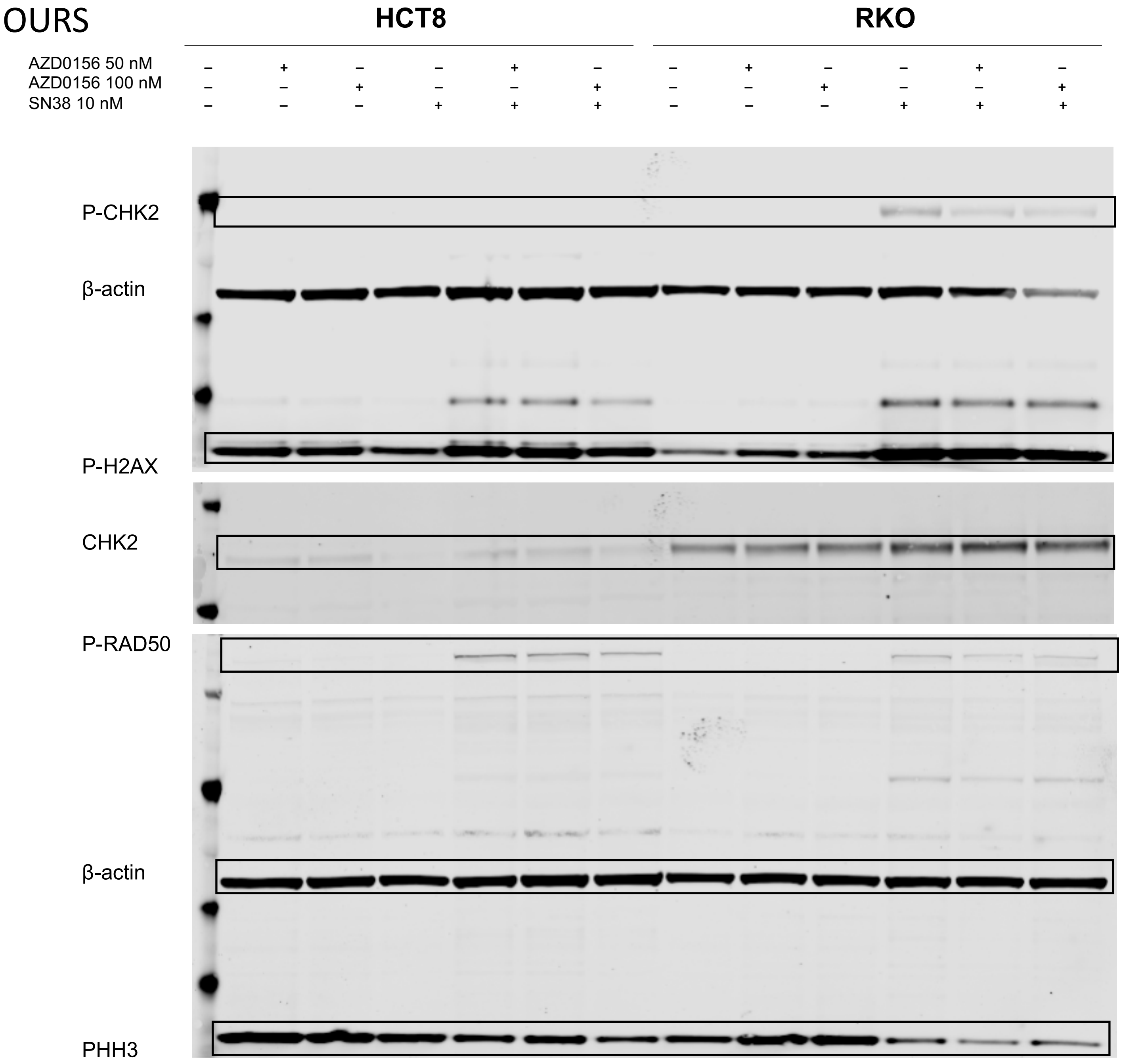

HCT8
RKO
AZD0156 50 nM
AZD0156 100 nM
SN38 10 nM
P-CHK2
β-actin
P-H2AX
CHK2
P-RAD50
β-actin
PHH3

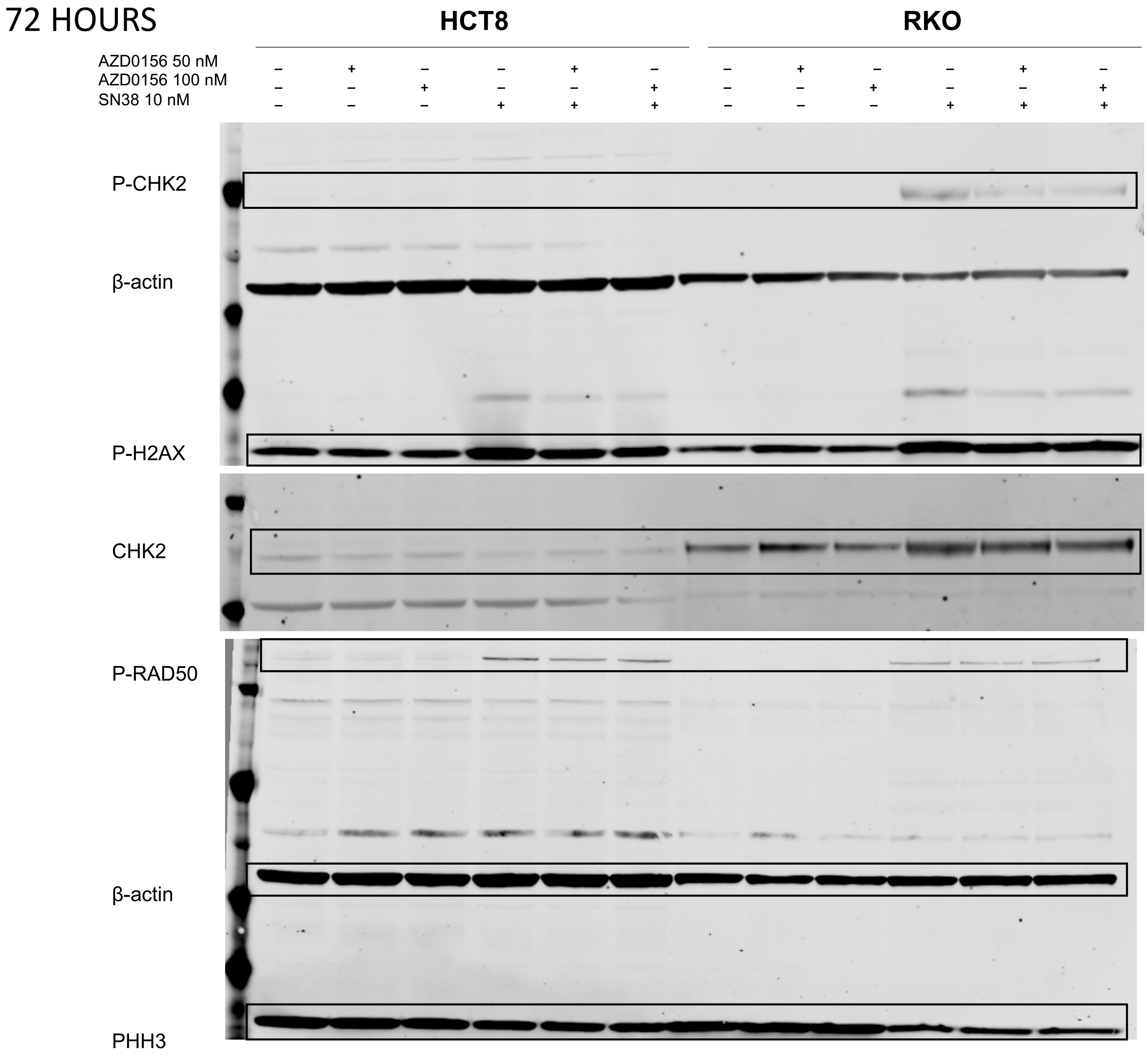
72 HOURS
HCT8
RKO
AZD0156 50 nM
AZD0156 100 nM
SN38 10 nM
P-CHK2
β-actin
P-H2AX
CHK2
P-RAD50
β-actin
PHH3

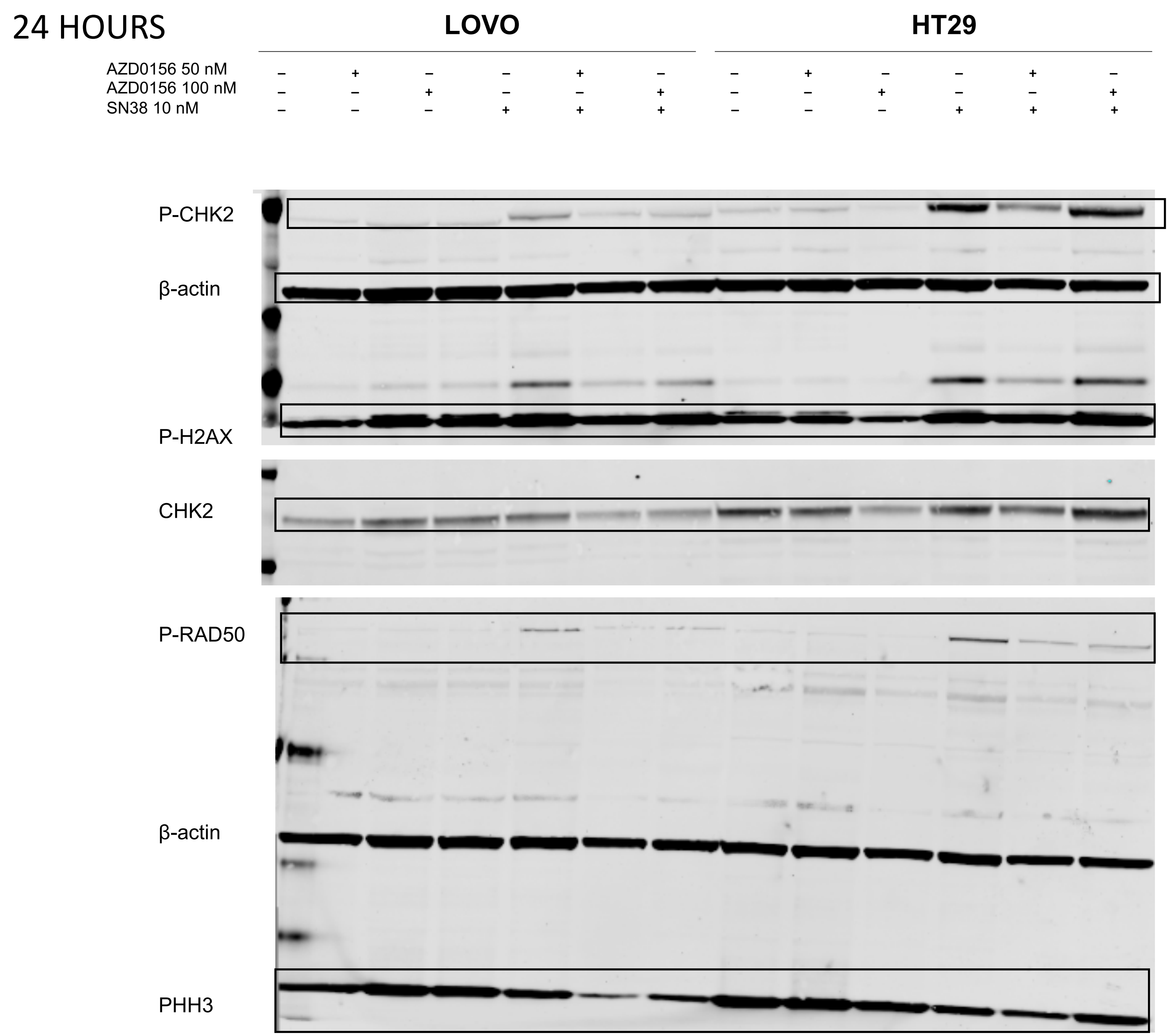

24 HOURS
LOVO
HT29
AZD0156 50 nM
AZD0156 100 nM
SN38 10 nM
P-CHK2
β-actin
P-H2AX
CHK2
P-RAD50
β-actin
PHH3

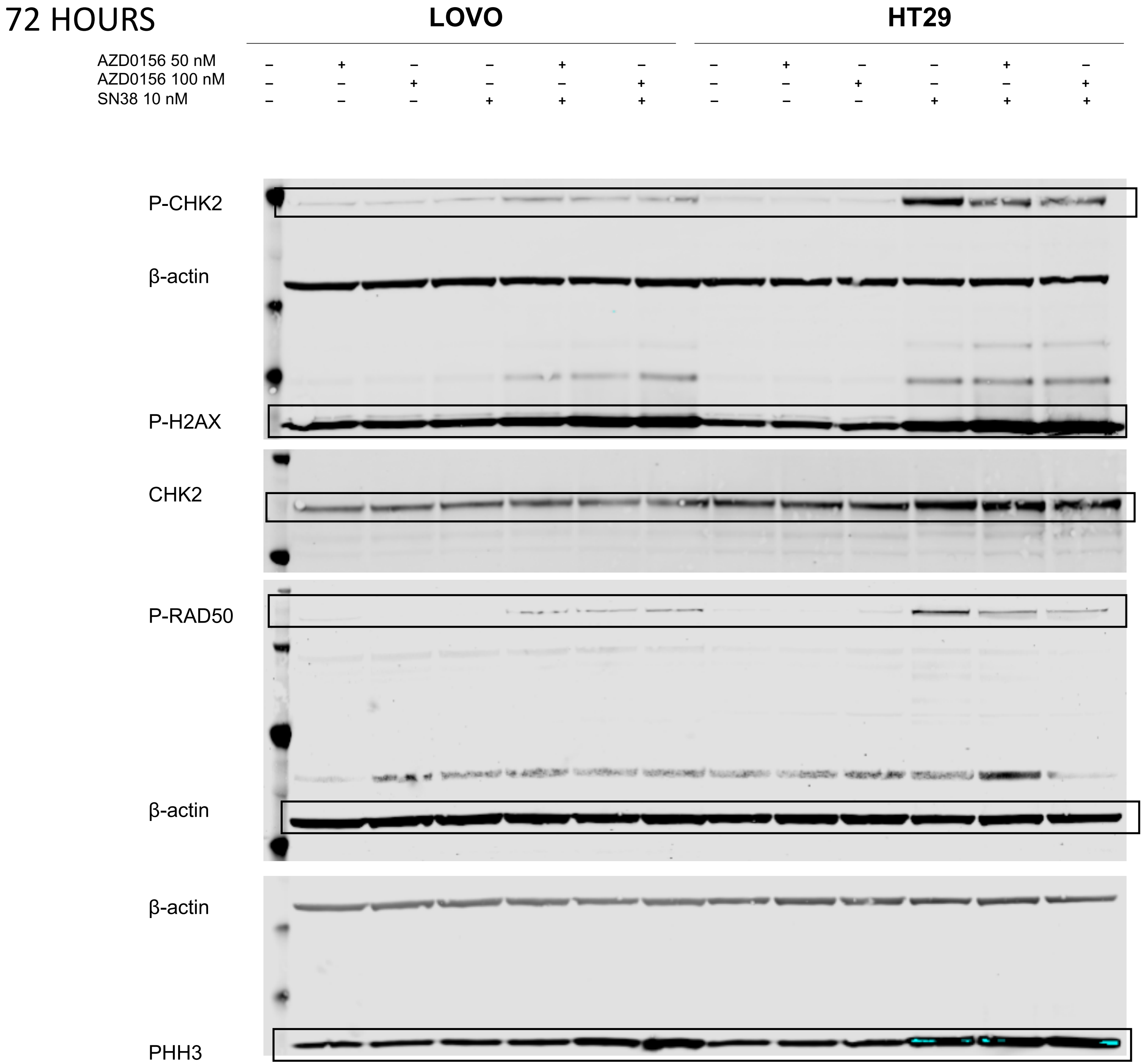
72 HOURS
LOVO
HT29
AZD0156 50 nM
AZD0156 100 nM
SN38 10 nM
P-CHK2
β-actin
P-H2AX
CHK2
P-RAD50
β-actin
β-actin
PHH3
